# Supplementary material for: TMEM55B links autophagy flux, lysosomal repair, and TFE3 activation in response to oxidative stress
Source: Nat Commun. 2024 Jan 2;15:93. doi: 10.1038/s41467-023-44316-6 (PMC10761734; doi:10.1038/s41467-023-44316-6)
Supplement: Supplementary file 1 — Supplementary Information [file 41467_2023_44316_MOESM1_ESM.pdf]

Supplementary information

TMEM55B links autophagy flux, lysosomal repair, and TFE3 activation in response to oxidative stress

Eutteum Jeong[1], Rose Willett[1], Alberto Rissone[1], Martina La Spina[1], and Rosa Puertollano[1*]

[1]Cell and Developmental Biology Center, National Heart, Lung, and Blood Institute, National Institutes of Health, Bethesda, MD, USA.

[*]Correspondence: Rosa Puertollano (puertolr@mail.nih.gov)

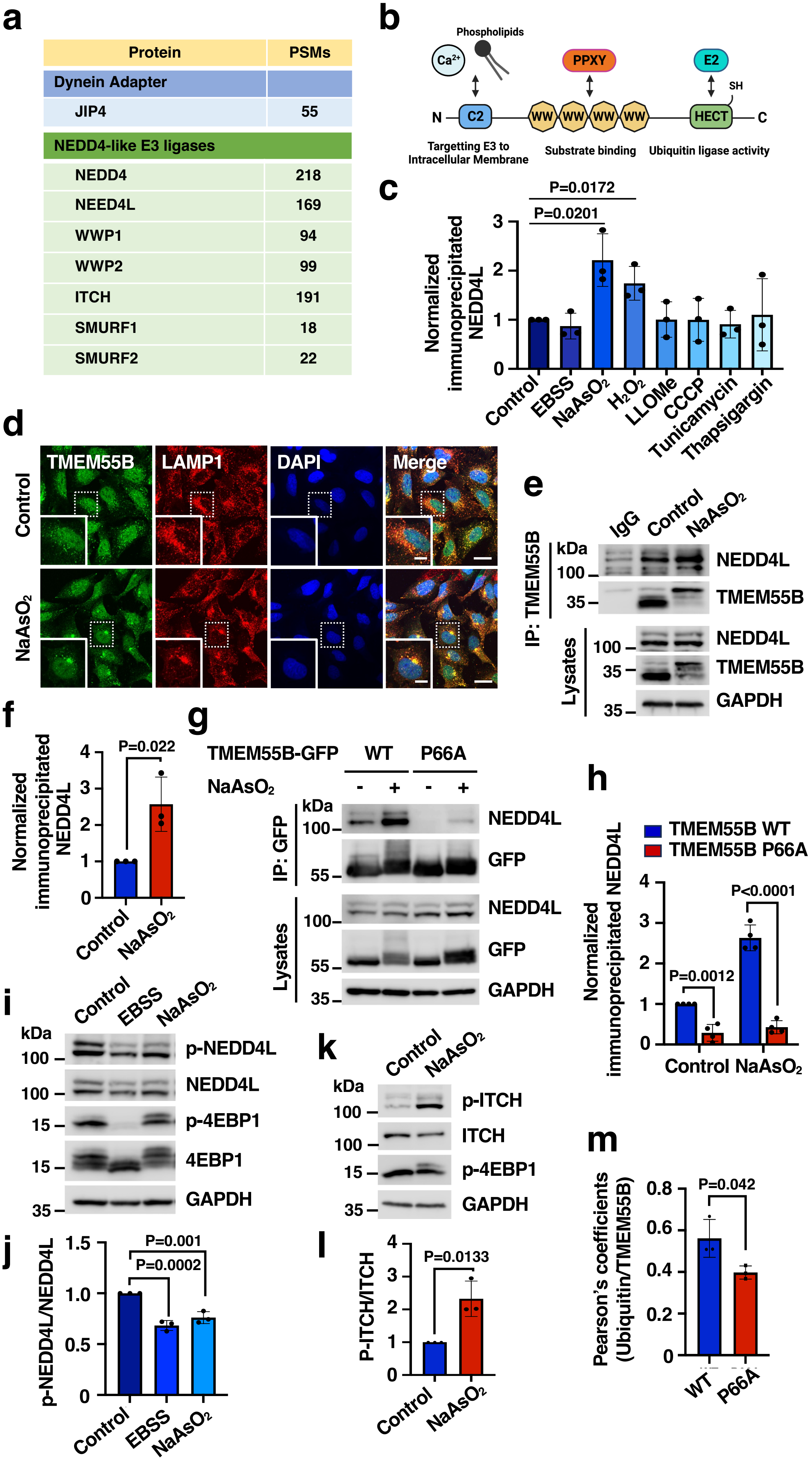
a
Protein
PSMs
Dynein Adapter
JIP4
55
NEDD4-like E3 ligases
NEDD4
218
NEED4L
169
WWP1
94
WWP2
99
ITCH
191
SMURF1
18
SMURF2
22
b
Phospholipids
Ca^{2+}
PPXY
E2
SH
N
C2
WW
WW
WW
WW
HECT
C
Targetting E3 to Intracellular Membrane
Substrate binding
Ubiquitin ligase activity
c
P=0.0172
P=0.0201
Normalized immunoprecipitated NEDD4L
Control
EBSS
$NaAsO_2$
H_2O_2
LLOMe
CCCP
Tunicamycin
Thapsigargin
d
TMEM55B
LAMP1
DAPI
Merge
Control
$NaAsO_2$
e
IgG
Control
$NaAsO_2$
IP: TMEM55B
Lysates
kDa
100
35
NEDD4L
TMEM55B
GAPDH
f
P=0.022
Normalized immunoprecipitated NEDD4L
Control
$NaAsO_2$
g
TMEM55B-GFP
WT
P66A
$NaAsO_2$
IP: GFP
Lysates
kDa
100
55
35
NEDD4L
GFP
GAPDH
h
TMEM55B WT
TMEM55B P66A
P<0.0001
P=0.0012
Normalized immunoprecipitated NEDD4L
Control
$NaAsO_2$
i
Control
EBSS
$NaAsO_2$
kDa
p-NEDD4L
NEDD4L
p-4EBP1
4EBP1
GAPDH
j
P=0.001
P=0.0002
p-NEDD4L/NEDD4L
Control
EBSS
$NaAsO_2$
k
Control
$NaAsO_2$
kDa
p-ITCH
ITCH
p-4EBP1
GAPDH
l
P=0.0133
P-ITCH/ITCH
Control
$NaAsO_2$
m
P=0.042
Pearson's coefficients (Ubiquitin/TMEM55B)
WT
P66A

Supplementary Figure 1. TMEM55B interacts with NEDD4-like E3 ligases under oxidative stress.

a, TMEM55B interactors identified by immunoprecipitation and mass spectrometry analysis. **b,** Schematic representation of domain composition of NEDD4-like E3 ligases. Illustration created with BioRender.com**. c,** Quantification of immunoblots shown in (Fig 1f). The data represent means ± SEM, n=3 independent experiments. Statistical significance was determined by using t-test. **d,** U2OS cells were treated with or without $NaAsO_2$ (300 μM) for 2 h. Cells were fixed and immunostained with antibodies against TMEM55B (green) and LAMP1 (red). DAPI staining is shown in blue. Scale bars, 20 μm. Inset scale bars, 10 μm. n=3. **e,** U2OS cells treated with or without $NaAsO_2$ (300 μM) for 2 h were lysed and immunoprecipitated with anti-TMEM55B antibody. **f,** Quantification of immunoblots shown in (**e**). The data represent means ± SEM, n=3 independent experiments. Statistical significance was determined by using t-test. **g,** U2OS cells infected with adenovirus expressing TMEM55B-GFP-WT or P66A were incubated with or without $NaAsO_2$ (300 μM) for 2 h. Cells were lysed and pulled down with GFP beads. **h,** Quantification of immunoblots shown in (**g**). The data represent means ± SEM, n=4 independent experiments. Statistical significance was determined by using two-way ANOVA with Sidak's multiple comparisons. **i,** U2OS cells incubated with either EBSS for 4 h or $NaAsO_2$ (300 μM) for 2 h were lysed and followed by western blotting. The results are representative of four independent experiments. **j,** Quantification of immunoblots shown in (**i**). The data represent means ± SEM, n=3 independent experiments Statistical significance was determined by using one-way ANOVA with Dunnett's multiple comparisons. **k,** U2OS cells incubated with or without $NaAsO_2$ (300 μM) for 2 h were lysed and followed by western blotting. **l,** Quantification of immunoblots shown in (**k**). The data represent means ± SEM, n=3 independent experiments. Statistical significance was determined by using t-test. **m,** Quantification of immunofluorescence images shown in (Fig. 1g) with Pearson's correlation coefficient. The data represent means ± SEM, n=3 independent experiments. Statistical significance was determined by using t-test. Source data are provided as a Source Data file.

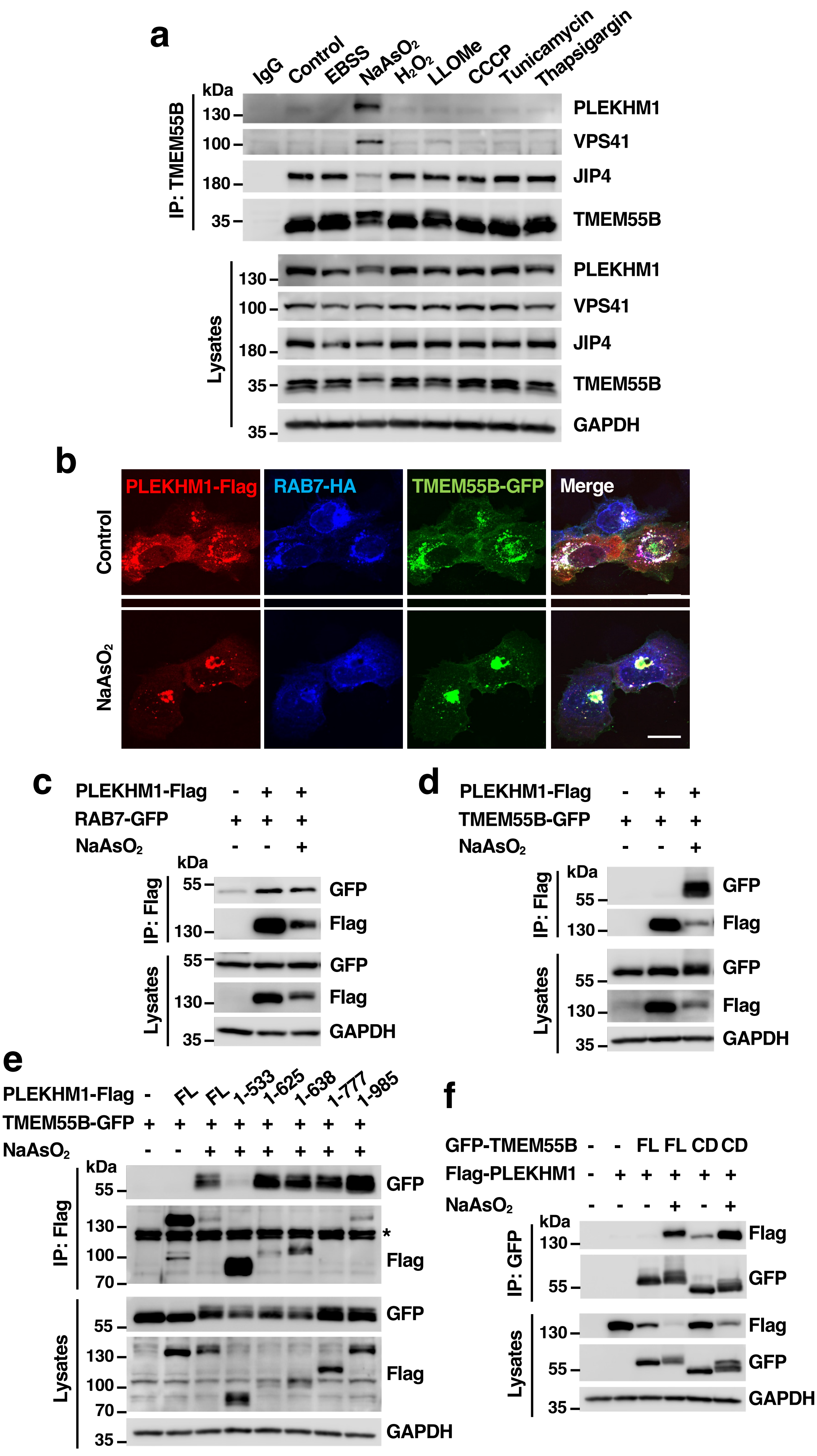

a
IgG
Control
EBSS
NaAsO2
H2O2
LLOMe
CCCP
Tunicamycin
Thapsigargin
kDa
IP: TMEM55B
Lysates
PLEKHM1
VPS41
JIP4
TMEM55B
GAPDH
b
PLEKHM1-Flag
RAB7-HA
TMEM55B-GFP
Merge
Control
NaAsO2
c
PLEKHM1-Flag
RAB7-GFP
NaAsO2
IP: Flag
GFP
Flag
GAPDH
d
TMEM55B-GFP
e
FL
1-533
1-625
1-638
1-777
1-985
*
f
GFP-TMEM55B
Flag-PLEKHM1
CD
IP: GFP

Supplementary Figure 2. PLEKHM1 interacts with TMEM55B through its PH1 domain under $NaAsO_2$ treatment.

a, U2OS cells treated with various drugs were lysed and immunoprecipitated with anti-TMEM55B antibody. EBSS for 4 h, $NaAsO_2$ (300 μM) for 2 h, H_2O_2 (500 μM) for 4 h, LLOMe (1 mM) for 2 h, CCCP (25 μM) for 4 h, Tunicamycin (10 μg/ml) for 4 h, Thapsigargin (10 μM) for 4 h. The results are representative of four independent experiments. **b,** U2OS cells transfected with plasmids encoding PLEKHM1-Flag, RAB7-HA and TMEM55B-GFP were treated with $NaAsO_2$ (300 μM) for 2 h. Cells were fixed and immunostained with antibodies against Flag (red) and HA (blue). TMEM55B-GFP is shown in green. Scale bars, 20 μm. n=3. **c-e,** U2OS cells transfected with plasmids encoding (**c**) PLEKHM1-Flag and RAB7-GFP, (**d**) PLEKHM1-Flag and TMEM55B-GFP, (**e**) PLEKHM1-Flag variants and TMEM55B-GFP, were treated with or without $NaAsO_2$ (300 μM) for 2 h. Cells were lysed and immunoprecipitated with Flag beads. The asterisk indicates a non-specific band, FL indicates Full length. The results are representative of three independent experiments. **f,** U2OS cells transfected with PLEKHM1-Flag and either TMEM55B-GFP-FL or TMEM55B-GFP-CD were treated with or without $NaAsO_2$ (300 μM) for 2 h. Cells were lysed and pulled down with GFP beads. The results are representative of two independent experiments. Source data are provided as a Source Data file.

a

TMEM55B Phospho site	TMEM55B-GFP	TMEM55B-GFP + $NaAsO_2$
Phospho [T111]	0.04%	1.66%
Phospho [S162]	6.98%	29.91%

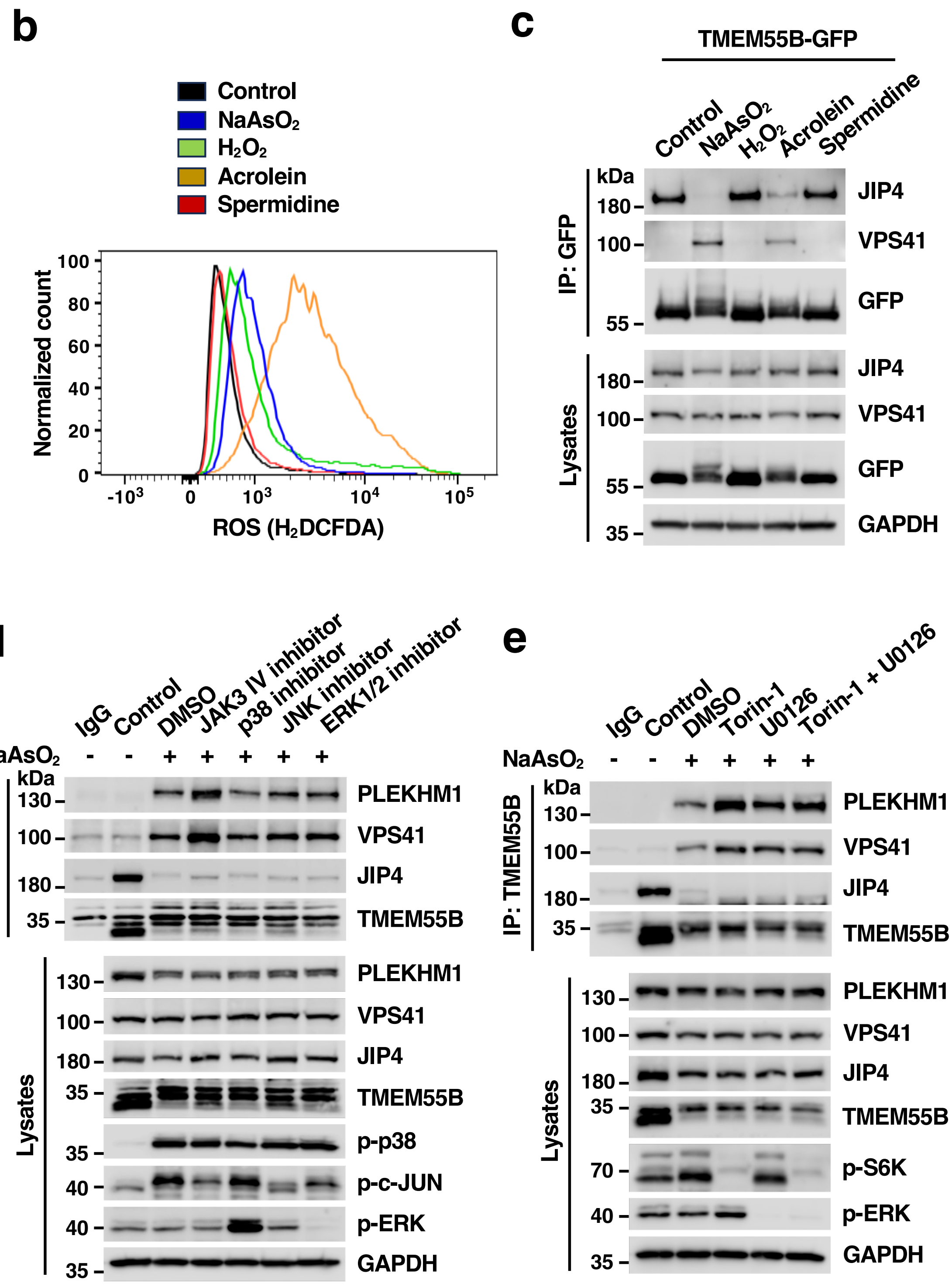

Supplementary Figure 3. TMEM55B phosphorylation in response to oxidative stress
a, Identification of phosphorylation sites in TMEM55B by mass spectrometry. U2OS cells were infected with adenovirus expressing TMEM55B-GFP-WT and either treated with $NaAsO_2$ (300 μM) or left untreated prior to immunoprecipitation and MS analysis. **b,** Intracellular ROS detection by FACS analysis in U2OS cells treated with $NaAsO_2$ (300 μM) for 2 h, H_2O_2 (500 μM) for 4 h, Acrolein (200 μM) for 4 h or Spermidine (300 μM) for 4 h. The results are representative of two independent experiments. **c,** U2OS cells were infected with adenovirus expressing TMEM55B-GFP-WT. Cells were then treated with $NaAsO_2$ (300 μM), H_2O_2 (500 μM), Acrolein (200 μM) or Spermidine (300 μM) for 4 h and pulled down with GFP beads. The results are representative of two independent experiments. **d,** U2OS cells were pre-treated with DMSO, JAK3 IV inhibitor (10 μM), p38 inhibitor: SB203580 (10 μM), JNK inhibitor: JNK Inhibitor VIII (10 μM) or ERK1/2 inhibitor: U0126 (10 μM) for 1 h and incubated together with $NaAsO_2$ (300 μM) for 2 h. Cells were lysed and immunoprecipitated with anti-TMEM55B antibody. The results are representative of three independent experiments. **e**, U2OS cells were pre-treated with DMSO, Torin-1 (250 nM) or U0126 (10 μM) for 30 min and incubated together with $NaAsO_2$ (300 μM) for 2 h. Cells were lysed and immunoprecipitated with anti-TMEM55B antibody. The results are representative of two independent experiments. Source data are provided as a Source Data file.

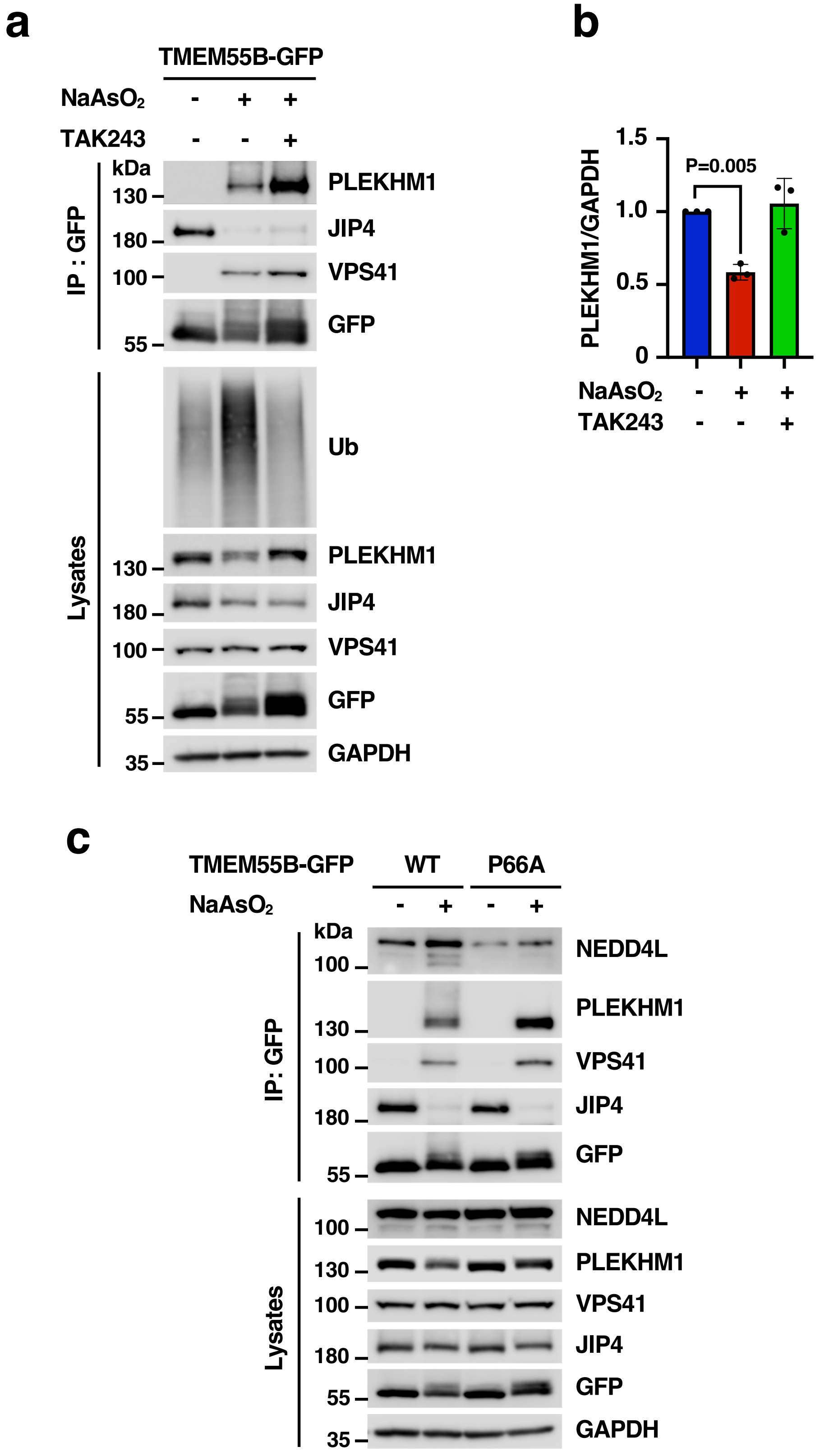

Supplementary Figure 4. $NaAsO_2$ induces ubiquitin-mediated PLEKHM1 degradation
a, U2OS cells infected with adenovirus expressing TMEM55B-GFP-WT were treated with or without TAK243 inhibitor for 6 h. Cells were then incubated with $NaAsO_2$ (300 μM) for 2h and pulled down with GFP beads. The results are representative of three independent experiments. **b,** Quantification of immunoblots shown in **(a)**. The data represent means ± SEM, n=3 independent experiments. Statistical significance was determined by using two-way ANOVA with Sidak's multiple comparisons. **c,** Hela cells infected with adenovirus expressing either TMEM55B-GFP-WT or P66A were treated with or without $NaAsO_2$ (300 μM) for 2 h. Cells were lysed and pulled down with GFP beads. The results are representative of two independent experiments. Source data are provided as a Source Data file.

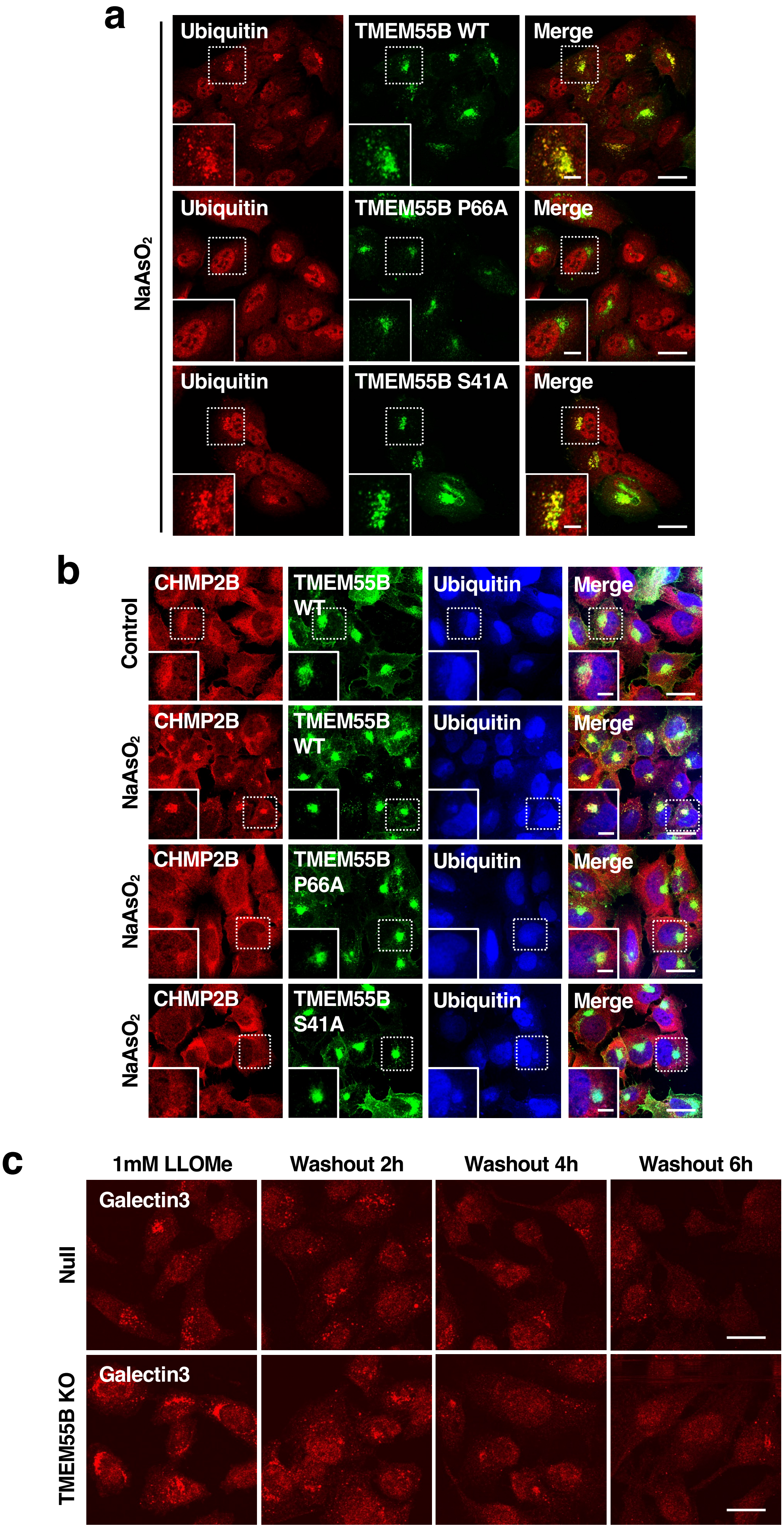
a
NaAsO$_2$
Ubiquitin
TMEM55B WT
Merge
Ubiquitin
TMEM55B P66A
Merge
Ubiquitin
TMEM55B S41A
Merge
b
Control
NaAsO$_2$
NaAsO$_2$
NaAsO$_2$
CHMP2B
TMEM55B WT
Ubiquitin
Merge
CHMP2B
TMEM55B WT
Ubiquitin
Merge
CHMP2B
TMEM55B P66A
Ubiquitin
Merge
CHMP2B
TMEM55B S41A
Ubiquitin
Merge
c
1mM LLOMe
Washout 2h
Washout 4h
Washout 6h
Null
TMEM55B KO
Galectin3
Galectin3

Supplementary Figure 5. TMEM55B facilitates ubiquitin dependent ESCRT complex recruitment.

a, U2OS TMEM55B KO cells transfected with plasmids encoding TMEM55B-GFP WT, P66A or S41A were treated with $NaAsO_2$ (300 μM) for 2 h. Cells were fixed and immunostained with antibody against ubiquitin (red). TMEM55B-GFP is shown in green. Scale bars, 20 μm. n=3. **b,** U2OS TMEM55B KO cells infected with adenovirus expressing TMEM55B-GFP WT, TMEM55B-GFP-P66A or TMEM55B-GFP-S41A were treated with or without $NaAsO_2$ (300 μM) for 2 h. Cells were fixed and immunostained with CHMP2B (red) and ubiquitin (blue) antibodies. Scale bars, 20 μm. n=3. **c,** Null or TMEM55B KO HeLa cells were treated with LLOMe (1 mM) for 2 h and followed by washout for the indicated times. Cells were fixed and immunostained with anti-Galectin3 antibody. Scale bars, 20 μm. n=3. Source data are provided as a Source Data file.

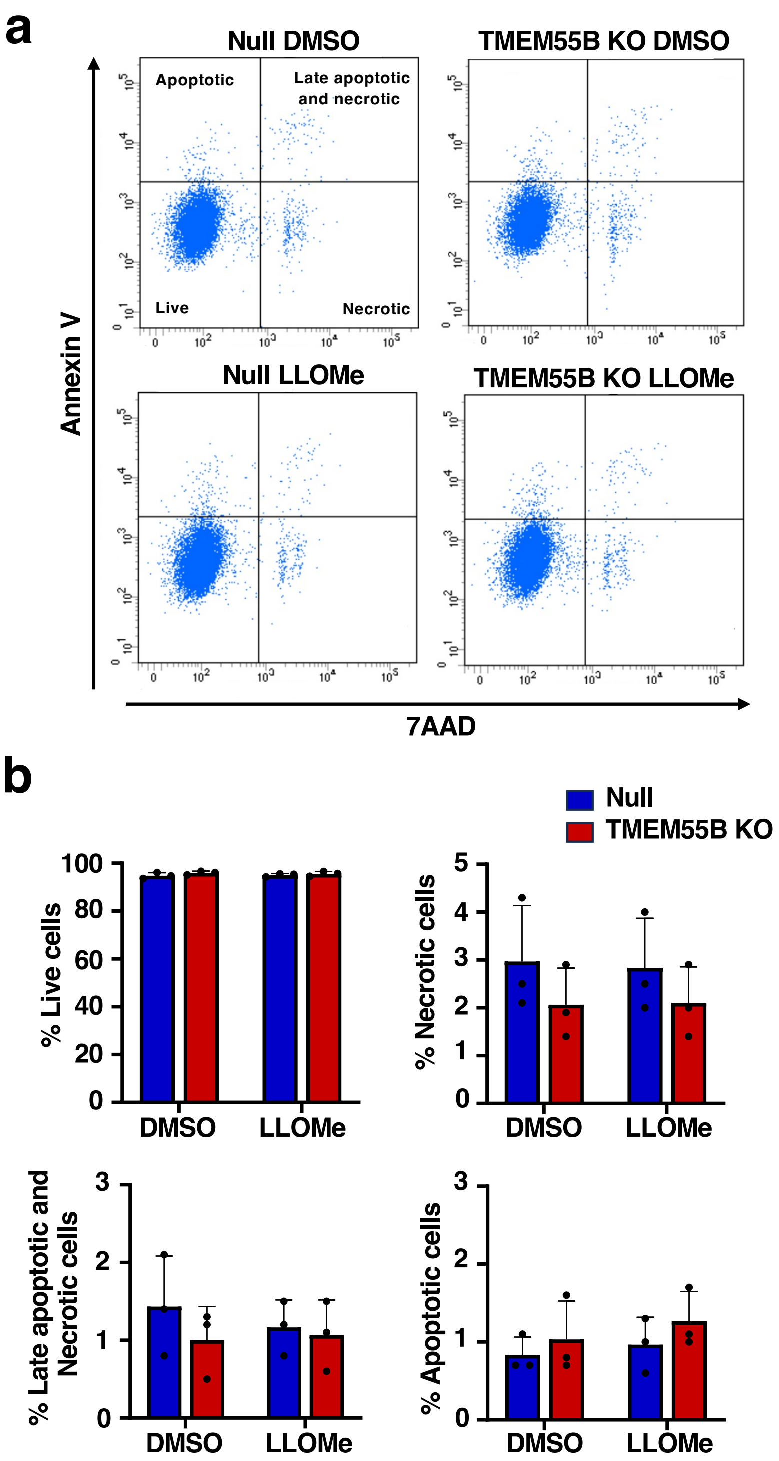

Supplementary Figure 6. LLOMe treatment has no effect on cell viability of TMEM55B KO cells. **a,** Null and TMEM55B KO U2OS cells were treated with DMSO or LLOMe (300 µM) for 10 h. Cells were analyzed by Flow cytometry with Annexin V and 7AAD. 7AAD+ are necrotic, Annexin V+ are apoptotic and Annexin V+/7AAD+ are late apoptotic and necrotic cells. **b,** Quantification of the population of live, necrotic (7AAD+), apoptotic cells (Annexin V+) and late apoptotic and necrotic cells (Annexin+/7AAD+) from (**a**). Data taken from three independent experiments and statistical significance was determined by using two-way ANOVA with Sidak's multiple comparisons. Source data are provided as a Source Data file.

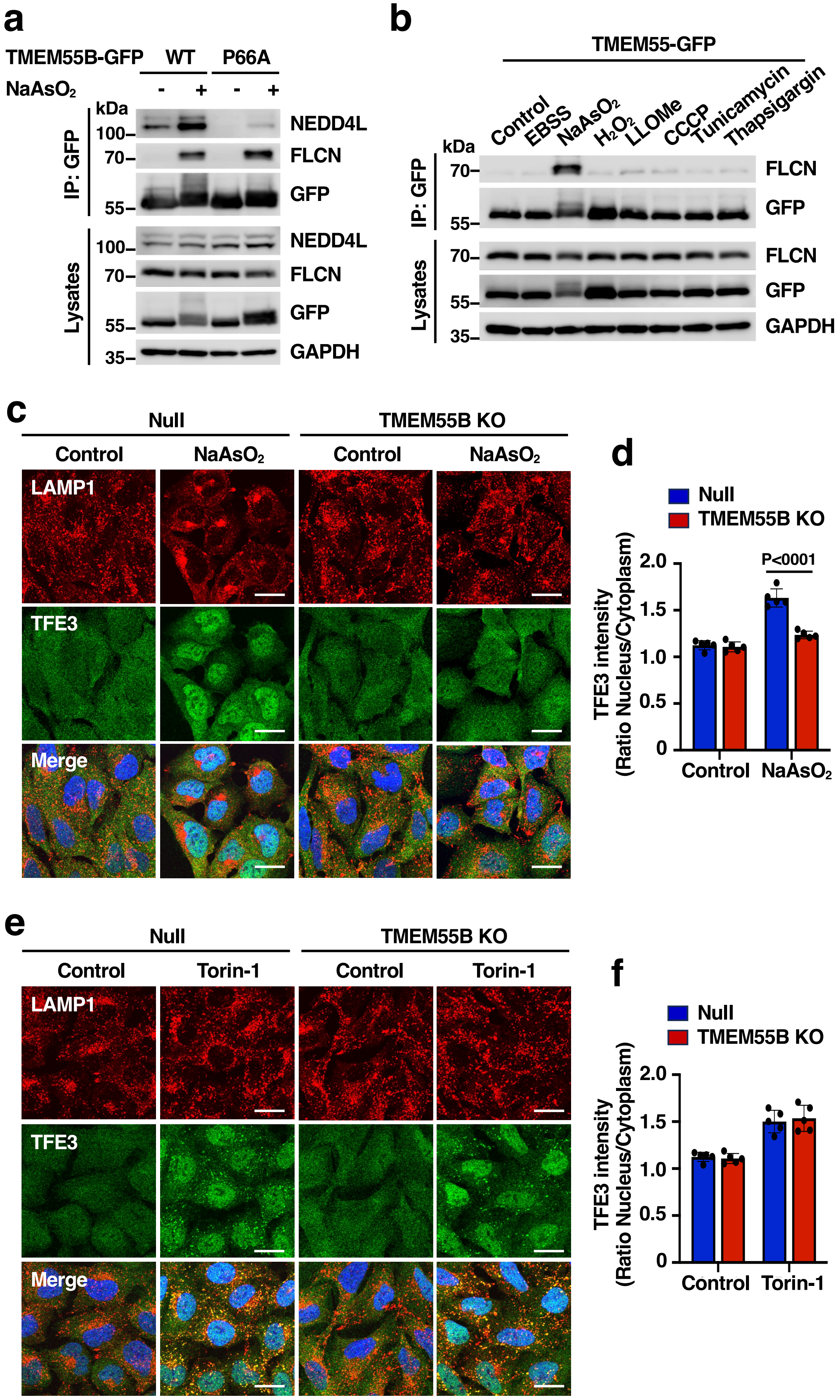

a
TMEM55B-GFP WT P66A
NaAsO2 - + - +
kDa
IP: GFP
Lysates
NEDD4L
FLCN
GFP
GAPDH
100
70
55
35
b
TMEM55-GFP
Control EBSS NaAsO2 H2O2 LLOMe CCCP Tunicamycin Thapsigargin
c
Null
TMEM55B KO
Control NaAsO2 Control NaAsO2
LAMP1
TFE3
Merge
d
Null
TMEM55B KO
TFE3 intensity (Ratio Nucleus/Cytoplasm)
P<0001
Control NaAsO2
e
Null
TMEM55B KO
Control Torin-1 Control Torin-1
LAMP1
TFE3
Merge
f
Null
TMEM55B KO
TFE3 intensity (Ratio Nucleus/Cytoplasm)
Control Torin-1

Supplementary Figure 7. TMEM55B is required for efficient TFE3 activation following $NaAsO_2$ treatment.

a, U2OS cells infected with adenovirus expressing TMEM55B-GFP-WT or P66A were treated with or without $NaAsO_2$ (300 μM) for 2 h. Cells were lysed and pulled down with GFP beads. The results are representative of three independent experiments. **b,** U2OS cells infected with adenovirus expressing TMEM55B-GFP-WT were treated with various drugs. Cells were lysed and pulled down with GFP beads. EBSS for 4 h, $NaAsO_2$ (300 μM) for 2 h, H_2O_2 (500 μM) for 4 h, LLOMe (1 mM) for 2 h, CCCP (25 μM) for 4 h, Tunicamycin (10 μg/ml) for 4 h, Thapsigargin (10 μM) for 4 h. The results are representative of three independent experiments. **c, e** Null and TMEM55B KO U2OS cells were treated with or without $NaAsO_2$ (300 μM) for 2 h (**c**) or Torin-1 (250 nM) for 2 h (**e**). Cells were fixed and immunostained with antibodies against LAMP1 (red) and TFE3 (green). Scale bars, 20 μm. n=3. **d, f** Quantification of immunofluorescence images shown in (**c**) and (**e**), respectively. The data represent means ± SEM, n>200 cells examined over 5 independent experiments. Statistical significance was determined by using two-way ANOVA with Sidak's multiple comparisons. Source data are provided as a Source Data file.

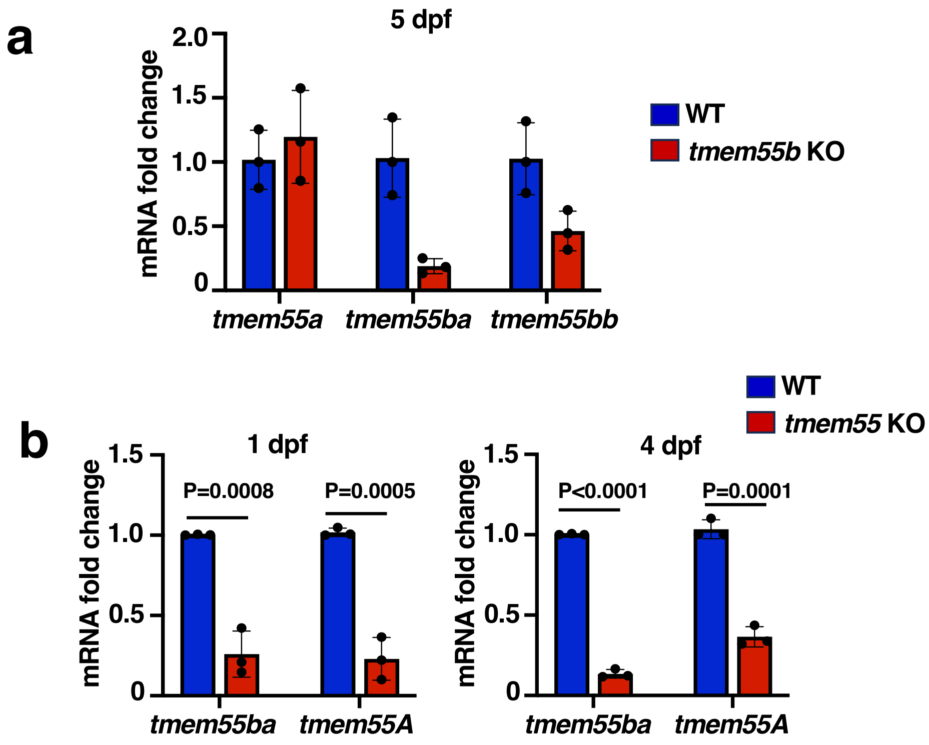

Supplementary Figure 8. Frame-shift mutations in *tmem55b*-KO and tmem55-KO mutants induce activation of Nonsense-mediated mRNA decay (NMD) mechanisms. a, Relative qPCR analysis showing expression levels of *tmem55a*, *tmem55ba* and *tmem55bb* in 5 dpf WT and the *tmem55b*-KO mutants. Error bars show the statistical variation of relative quantity (RQ) calculated using a 99% confidence level. The result show one representative experiment of three independent experiments. **b**, Relative qPCR analysis showing expression levels of *tmem55ba* and *tmem55a* in WT and *tmem55*-KO embryos at 1 and 4 dpf. Data represented as geometric mean ± SD and significance tested with Student's t-test from at least three independent experiments. Source data are provided as a Source Data file.

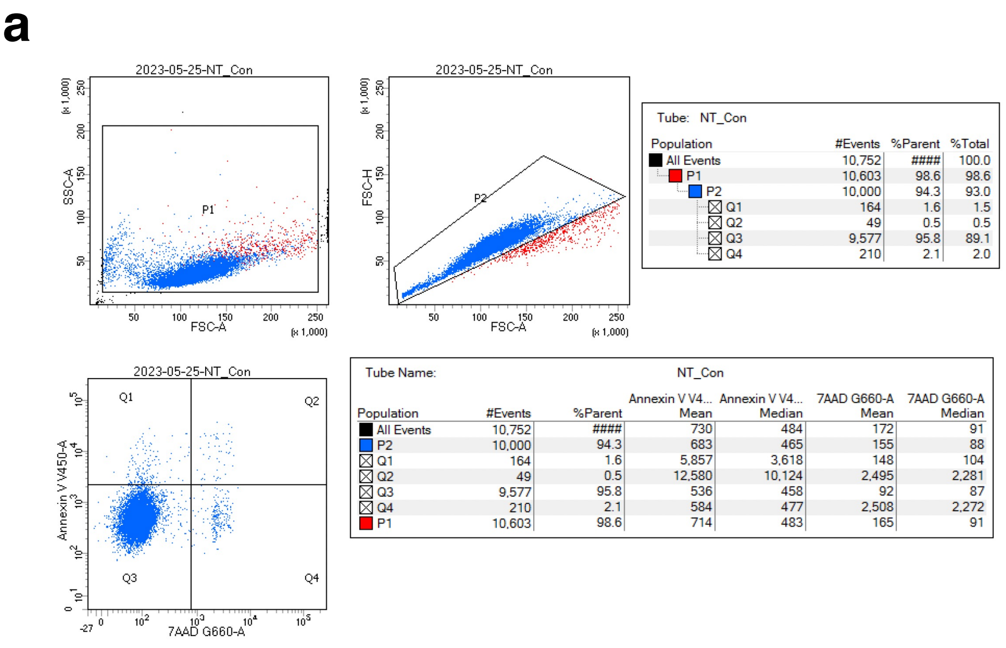

Tube: NT_Con

Population	#Events	%Parent	%Total
All Events	10,752	####	100.0
P1	10,603	98.6	98.6
P2	10,000	94.3	93.0
Q1	164	1.6	1.5
Q2	49	0.5	0.5
Q3	9,577	95.8	89.1
Q4	210	2.1	2.0

Tube Name: NT_Con

Population	#Events	%Parent	Annexin V V4... Mean	Annexin V V4... Median	7AAD G660-A Mean	7AAD G660-A Median
All Events	10,752	####	730	484	172	91
P2	10,000	94.3	683	465	155	88
Q1	164	1.6	5,857	3,618	148	104
Q2	49	0.5	12,580	10,124	2,495	2,281
Q3	9,577	95.8	536	458	92	87
Q4	210	2.1	584	477	2,508	2,272
P1	10,603	98.6	714	483	165	91

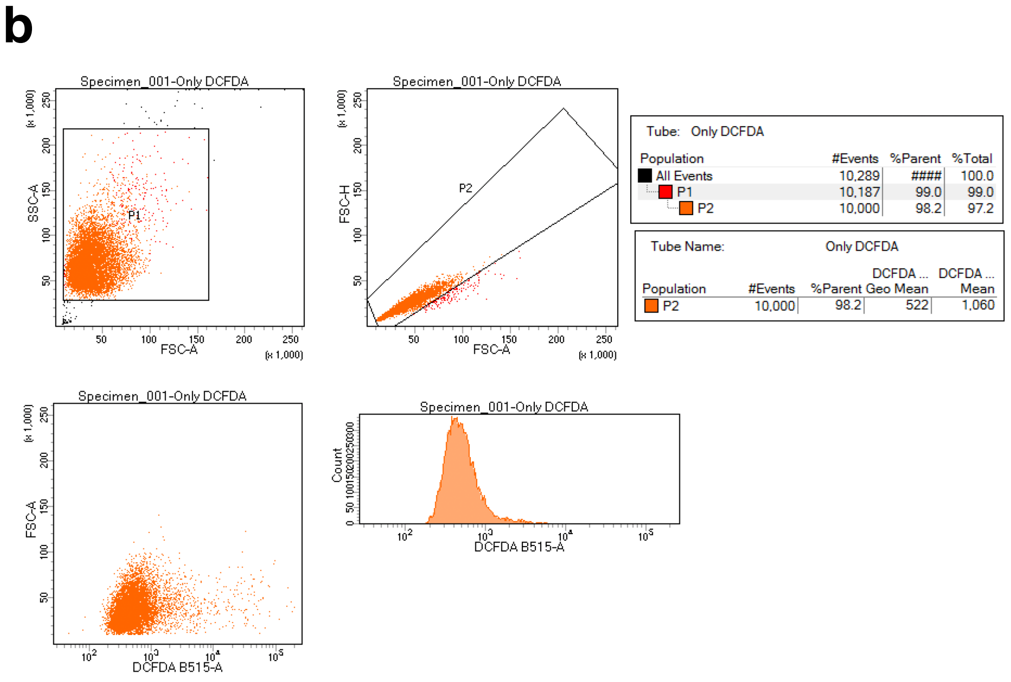

Tube: Only DCFDA

Population	#Events	%Parent	%Total
All Events	10,289	####	100.0
P1	10,187	99.0	99.0
P2	10,000	98.2	97.2

Tube Name: Only DCFDA

Population	#Events	%Parent	DCFDA ... Geo Mean	DCFDA ... Mean
P2	10,000	98.2	522	1,060

Supplementary Figure 9. FACS gating strategy. Examples of the gating strategy used for Annexin V and 7-AAd staining (**a**) and ROS production measurement (**b**).

Supplementary Table 1. List of primers and gRNAs used in this study

gRNAs

tmem55ba	*tmem55ba* exon2 gRNA atttaggtgacactataGACGTCAGGGGGGAATATGGgttttagagctagaaatagc
tmem55bb	*tmem55bb* exon2 gRNA taatacgactcactataGGAGAGCCCAGCGGGGAGTAgttttagagctagaaatagc
tmem55a	*tmem55a* exon1 gRNA taatacgactcactataGGCGTGACGTTCCCCGAGTTgttttagagctagaaatagc
	tmem55a exon4 gRNA taatacgactcactataGGATCACCATCACTGGGCTCgttttagagctagaaatagc

NT oligo2
AAAAGCACCGACTCGGTGCCACTTTTTCAAGTTGATAACGGACTAGCCTTATTTTAACTTGCTATTTCTAGCTCTA
AAAC

gDNA genotyping primers

tmem55ba	*tmem55ba* exon2 F	GTGTCTTCGTTTCCGAGCTCAACTCA
	tmem55ba exon2 R	TGTAAAACGACGGCCAGTGCACTTGACCACATGTTGATG
tmem55bb	*tmem55bb* exon2 F	GTGTCTTGTCAGTCATTTTCCCCCAGA
	tmem55bb exon2 R	TGTAAAACGACGGCCAGTcTGTAGCCTCATTGCAGACG
tmem55a	*tmem55a* exon1 F	GTGTCTTTTTTTTCTGTGCCGATCTCCT
	tmem55a exon1 R	TGTAAAACGACGGCCAGTCCGTCAATCTCGGTTACCTC
	tmem55a exon1 F	GTGTCTTCTGCTGCTGCTGTTTCTGTT
	tmem55a exon4 F	GTGTCTTCGCTGACGTTGTTTGTATGG
	tmem55a exon4 R	TGTAAAACGACGGCCAGTAAGGAATGTGTTTCCGCAG
	tmem55a exon4 F	GTGTCTTATTTCGAGACCTTTCTTGTATCAG

mRNA sequencing primers

tmem55ba	55ba-exon2 mRNA F	TGTAAAACGACGGCCAGTCAAACCGCAGAGTTTTCCTC
	55ba-exon2 mRNA R	GTGTCTTGCCTGCAGGAGCATTCTTTA
tmem55bb	55bb-exon2 mRNA F	TGTAAAACGACGGCCAGTCTACCGACCGATCAGCAAAC
	55bb-exon2 mRNA R	GTGTCTTGCAGTTACACGGACACCTCA
tmem55a	55a-exon1 mRNA F	GTGTCTTTTTTTTCTGTGCCGATCTCCT
	55a-exon1 mRNA R	TGTAAAACGACGGCCAGTGACTGGCAATGGCTGTGTAG
	55a-exon4 mRNA F	GTGTCTTTGCAACTGCCTGCTAATCTG
	55a-exon4 mRNA R	TGTAAAACGACGGCCAGTGCTCCATCCAAAGGAATGTG

qPCR primers

tmem55ba	*tmem55ba* qPCR F	ATATCCAAGGAGGCGGAGTC
	tmem55ba qPCR R	AGTCAACACCACCAGCAACA
tmem55bb	*tmem55bb* qPCR F	CAGAGGTACCCTCGAAAACG
	tmem55bb qPCR R	TAACCAGCACCAGGACAATG
tmem55a	*tmem55a* qPCR F	GGCAGTGCTTTACCCAGAAG
	tmem55a qPCR R	TAATGGCTCCCCAATAGCAG
