# Supplementary material for: TMEM55B links autophagy flux, lysosomal repair, and TFE3 activation in response to oxidative stress
Source: Nat Commun. 2024 Jan 2;15:93. doi: 10.1038/s41467-023-44316-6 (PMC10761734; doi:10.1038/s41467-023-44316-6)
Supplement: Supplementary file 3 — Description of Additional Supplementary Files [file 41467_2023_44316_MOESM3_ESM.pdf]

Description of Additional Supplementary Files

File Name: **Supplementary Data 1**
Description: Identification of TMEM55B binding partners by mass spectrometry analysis. The data relate to Figures 2a, 5a, and Supplementary Figure 1a.

File Name: **Supplementary Data 2**
Description: Identification of ubiquitination sites in TMEM55B by mass spectrometry analysis. The data relate to Figure 1h.

File Name: **Supplementary Data 3**
Description: Identification of TMEM55B phosphorylation sites in untreated cells by mass spectrometry analysis. The data relate to Supplementary Figure 3a.

File Name: **Supplementary Data 4**
Description: Identification of TMEM55B phosphorylation sites in $NaAsO_2$-treated cells by mass spectrometry analysis. The data relate to Supplementary Figure 3a.
