# Supplementary material for: TMEM55B links autophagy flux, lysosomal repair, and TFE3 activation in response to oxidative stress
Source: Nat Commun. 2024 Jan 2;15:93. doi: 10.1038/s41467-023-44316-6 (PMC10761734; doi:10.1038/s41467-023-44316-6)
Supplement: Supplementary file 8 — Reporting Summary [file 41467_2023_44316_MOESM8_ESM.pdf]

nature portfolio

Corresponding author(s): Rosa Puertollano

Last updated by author(s): 11/27/2023

Reporting Summary

Nature Portfolio wishes to improve the reproducibility of the work that we publish. This form provides structure for consistency and transparency in reporting. For further information on Nature Portfolio policies, see our Editorial Policies and the Editorial Policy Checklist.

Statistics

For all statistical analyses, confirm that the following items are present in the figure legend, table legend, main text, or Methods section.

n/a	Confirmed	
☐	☒	The exact sample size (*n*) for each experimental group/condition, given as a discrete number and unit of measurement
☐	☒	A statement on whether measurements were taken from distinct samples or whether the same sample was measured repeatedly
☐	☒	The statistical test(s) used AND whether they are one- or two-sided<br>*Only common tests should be described solely by name; describe more complex techniques in the Methods section.*
☐	☒	A description of all covariates tested
☐	☒	A description of any assumptions or corrections, such as tests of normality and adjustment for multiple comparisons
☐	☒	A full description of the statistical parameters including central tendency (e.g. means) or other basic estimates (e.g. regression coefficient) AND variation (e.g. standard deviation) or associated estimates of uncertainty (e.g. confidence intervals)
☐	☒	For null hypothesis testing, the test statistic (e.g. *F*, *t*, *r*) with confidence intervals, effect sizes, degrees of freedom and *P* value noted<br>*Give P values as exact values whenever suitable.*
☒	☐	For Bayesian analysis, information on the choice of priors and Markov chain Monte Carlo settings
☒	☐	For hierarchical and complex designs, identification of the appropriate level for tests and full reporting of outcomes
☐	☒	Estimates of effect sizes (e.g. Cohen's *d*, Pearson's *r*), indicating how they were calculated

Our web collection on statistics for biologists contains articles on many of the points above.

Software and code

Policy information about availability of computer code

Data collection	Immunofluorescence images were obtained from LSM 510 Meta confocal microscope and immunoblot images were obtained from Amersham Imager 600. Quantitative real-time PCR was performed by QuantStudio™ 12K Flex Real-Time PCR System (Applied Biosystems) and primers were designed with Primer3web (version 4.1.0). Flow Cytometry was performed with BD LSR Fortessa.
Data analysis	NAImage analysis was performed by ImageJ (FIJI) version 2.1.0/1.53c. Flow Cytometry data was analyzed with BD FACSDiva. Statistical analysis was performed with Microsoft Excel 2019, Imaris x64 9.9.0. and GraphPad Prism 9.

For manuscripts utilizing custom algorithms or software that are central to the research but not yet described in published literature, software must be made available to editors and reviewers. We strongly encourage code deposition in a community repository (e.g. GitHub). See the Nature Portfolio guidelines for submitting code & software for further information.

Data

Policy information about availability of data

All manuscripts must include a data availability statement. This statement should provide the following information, where applicable:

- Accession codes, unique identifiers, or web links for publicly available datasets
- A description of any restrictions on data availability
- For clinical datasets or third party data, please ensure that the statement adheres to our policy

The authors declare that the data supporting the findings of this study are available within the manuscript and source data are provided with this paper.

The mass spectrometry proteomics data generated in this study have been deposited to the ProteomeXchange Consortium via the PRIDE partner repository with the dataset identifier PXD043609 and PXD046394.

Research involving human participants, their data, or biological material

Policy information about studies with human participants or human data. See also policy information about sex, gender (identity/presentation), and sexual orientation and race, ethnicity and racism.

Reporting on sex and gender	NA
Reporting on race, ethnicity, or other socially relevant groupings	NA
Population characteristics	NA
Recruitment	NA
Ethics oversight	NA

Note that full information on the approval of the study protocol must also be provided in the manuscript.

Field-specific reporting

Please select the one below that is the best fit for your research. If you are not sure, read the appropriate sections before making your selection.

☒ Life sciences ☐ Behavioural & social sciences ☐ Ecological, evolutionary & environmental sciences

For a reference copy of the document with all sections, see nature.com/documents/nr-reporting-summary-flat.pdf

Life sciences study design

All studies must disclose on these points even when the disclosure is negative.

Sample size	No statistical methods were used to predetermined sample size. Sample size was chosen based on previous study used the similar methods
Data exclusions	No data were excluded from the analyses.
Replication	At least three independent experiments were performed to verify the reproducibility of the experimental findings. All experiments were reliably reproduced. All of the replication information are stated in figure legends.
Randomization	The experiments were not randomized
Blinding	The Investigators were not blinded to allocation during experiments and outcome assessment

Reporting for specific materials, systems and methods

We require information from authors about some types of materials, experimental systems and methods used in many studies. Here, indicate whether each material, system or method listed is relevant to your study. If you are not sure if a list item applies to your research, read the appropriate section before selecting a response.

Materials & experimental systems

n/a	Involved in the study	
☐	☒	Antibodies
☐	☒	Eukaryotic cell lines
☒	☐	Palaeontology and archaeology
☐	☒	Animals and other organisms
☒	☐	Clinical data
☒	☐	Dual use research of concern
☒	☐	Plants

Methods

n/a	Involved in the study	
☒	☐	ChIP-seq
☐	☒	Flow cytometry
☒	☐	MRI-based neuroimaging

Antibodies

Antibodies used	anti-TFE3 (Sigma, HPA023881), anti-phospho S321 TFE3 (YenZym Antibodies), anti-Flag (clone M2, Sigma-Aldrich, F1804), anti-LAMP1 from the Developmental Studies Hybridoma Bank deposited by August, J.T. (DSHB, 1D4B), anti-TMEM55B (Proteintech, 23992-1-AP), anti-CHMP2B (Proteintech, 12527-1-AP), anti-CHMP4B (Proteintech, 13683-1-AP), anti-TSG101 (Proteintech, 14497-1-AP), anti-

GAPDH (Santa Cruz Biotechnology, sc-365062), anti-Ub (Santa Cruz Biotechnology, sc-8017), anti-VPS41 (Santa Cruz biotechnology, sc-377271), anti-Galectin3 (Santa Cruz Biotechnology, sc-32790), anti-phospho-ITCH (Sigma-Aldrich, AB10050), anti-ITCH (Cell signaling Technology, 12117), anti-PLEKHM1 (Cell signaling Technology, 77092), anti-NEDD4 (Cell signaling Technology, 2740), anti-NEDD4L (Cell signaling Technology, 4013), anti-JIP4 (Cell signaling Technology, 5519), anti-LC3B (Cell signaling Technology, 43566), anti-phospho-p70 S6 Kinase (Cell Signaling Technology, 9205), anti-p70 S6 Kinase (Cell Signaling Technology, 2708), anti-phospho-4E-BP1 (Cell Signaling Technology, 2855), anti-4E-BP1 (Cell Signaling Technology, 9644), anti-phospho-p38 (Cell Signaling Technology, 4511), anti-phospho-c-Jun (Cell signaling Technology, 3270), anti-phospho-ERK (Cell signaling Technology, 9101), anti-LAMTOR1 (Cell Signaling Technology, 8975), anti-FNIP1 (Cell signaling Technology, 36892), anti-FLCN (Cell Signaling Technology, 3697), HRP-conjugated anti-rabbit IgG (Cell Signaling Technology, 7074), HRP-conjugated anti-mouse (Cell Signaling Technology, 7076), Alexa Fluor 568-conjugated goat anti-mouse IgG (Invitrogen, A21090), Alexa Fluor 488-conjugated goat anti-rabbit IgG (Invitrogen, A-11008) Alexa Fluor 488-conjugated goat anti-mouse IgG (Invitrogen, A-11001). For immunoblotting, antibodies were diluted as 1:1000. For immunofluorescence, antibodies were diluted from 1:50 to 1:1000.

Validation

anti-phospho S321 TFE3 (YenZym Antibodies) is validated in our previous paper ; https://doi.org/10.1080/15548627.2022.2029671
Validation information from manufacturer's for the other antibodies as below.
anti-TFE3 (Sigma, HPA023881) ; https://www.sigmaaldrich.com/US/en/product/sigma/hpa023881?gclid=CjwKCAiAsIGrBhAAEiwAEzMlC5GZu0iSV_nsrSNiCmYWPORI0DIgKutOV81ON_ZtG-JA9LAwxMX07RoC-IYQAvD_BwE
anti-Flag (clone M2, Sigma-Aldrich, F1804) ; https://www.sigmaaldrich.com/US/en/product/sigma/f1804?gclid=CjwKCAiAsIGrBhAAEiwAEzMlCw8ufwETbxK7VW4Cc-ySOxd9NBjO4vpnC_Phg72ng36V53oYkwf9axoCRy8QAvD_BwE
anti-LAMP1 from the Developmental Studies Hybridoma Bank deposited by August, J.T. (DSHB, 1D4B) ; https://dshb.biology.uiowa.edu/1D4B
anti-TMEM55B (Proteintech, 23992-1-AP) ; https://www.ptglab.com/products/TMEM55B-Antibody-23992-1-AP.htm
anti-CHMP2B (Proteintech, 12527-1-AP) ; https://www.ptglab.com/products/CHMP2B-Antibody-12527-1-AP.htm
anti-CHMP4B (Proteintech, 13683-1-AP) ; https://www.ptglab.com/products/CHMP4B-Antibody-13683-1-AP.htm
anti-TSG101 (Proteintech, 14497-1-AP) ; https://www.ptglab.com/products/TSG101-Antibody-14497-1-AP.htm
anti-GAPDH (Santa Cruz Biotechnology, sc-365062) ; https://www.scbt.com/p/gapdh-antibody-g-9?gad_source=1&gclid=CjwKCAiAsIGrBhAAEiwAEzMlCyJdpmHiphICGMeA4qjuYfTtuth4URePDUIO96OtA9t7hZ6VK_wjixoCAQ8QAvD_BwE
anti-Ub (Santa Cruz Biotechnology, sc-8017) ; https://www.scbt.com/p/ubiquitin-antibody-p4d1?gad_source=1&gclid=CjwKCAiAsIGrBhAAEiwAEzMlC7QEjkGEWa2pWS1ANyyNPgtgX8TfT7tjXEKvNYzNqObYR8eRTCMUrBoCA70QAvD_BwE
anti-VPS41 (Santa Cruz biotechnology, sc-377271) ; https://www.scbt.com/p/vps41-antibody-e-10
anti-Galectin3 (Santa Cruz Biotechnology, sc-32790) ; https://www.scbt.com/p/galectin-3-antibody-b2c10
anti-phospho-ITCH (Sigma-Aldrich, AB10050) ; https://www.sigmaaldrich.com/US/en/product/mm/ab10050
anti-ITCH (Cell signaling Technology, 12117) ; https://www.cellsignal.com/products/primary-antibodies/itch-d8q6d-rabbit-mab/12117
anti-PLEKHM1 (Cell signaling Technology, 77092) ; https://www.cellsignal.com/products/primary-antibodies/plekhm1-e9s3q-rabbit-mab/77092
anti-NEDD4 (Cell signaling Technology, 2740) ; https://www.cellsignal.com/products/primary-antibodies/nedd4-antibody/2740
anti-NEDD4L (Cell signaling Technology, 4013) https://www.cellsignal.com/products/primary-antibodies/nedd4l-antibody/4013
anti-JIP4 (Cell signaling Technology, 5519) ; https://www.cellsignal.com/products/primary-antibodies/jip4-spag9-d72f4-xp-rabbit-mab/5519
anti-LC3B (Cell signaling Technology, 43566) ; https://www.cellsignal.com/products/primary-antibodies/lc3b-e7x4s-xp-rabbit-mab/43566
anti-phospho-p70 S6 Kinase (Cell Signaling Technology, 9205) ; https://www.cellsignal.com/products/primary-antibodies/phospho-p70-s6-kinase-thr389-antibody/9205
anti-p70 S6 Kinase (Cell Signaling Technology, 2708) ; https://www.cellsignal.com/products/primary-antibodies/p70-s6-kinase-49d7-rabbit-mab/2708
anti-phospho-4E-BP1 (Cell Signaling Technology, 2855) ; https://www.cellsignal.com/products/primary-antibodies/phospho-4e-bp1-thr37-46-236b4-rabbit-mab/2855
anti-4E-BP1 (Cell Signaling Technology, 9644) ; https://www.cellsignal.com/products/primary-antibodies/4e-bp1-53h11-rabbit-mab/9644
anti-phospho-p38 (Cell Signaling Technology, 4511) ; https://www.cellsignal.com/products/primary-antibodies/phospho-p38-mapk-thr180-tyr182-d3f9-xp-rabbit-mab/4511
anti-phospho-c-Jun (Cell signaling Technology, 3270) ; https://www.cellsignal.com/products/primary-antibodies/phospho-c-jun-ser73-d47g9-xp-rabbit-mab/3270
anti-phospho-ERK (Cell signaling Technology, 9101) ; https://www.cellsignal.com/products/primary-antibodies/phospho-p44-42-mapk-erk1-2-thr202-tyr204-antibody/9101
anti-LAMTOR1 (Cell Signaling Technology, 8975) ; https://www.cellsignal.com/products/primary-antibodies/lamtor1-c11orf59-d11h6-xp-rabbit-mab/8975
anti-FNIP1 (Cell signaling Technology, 36892) ; https://www.cellsignal.com/products/primary-antibodies/fnip1-e9y5u-rabbit-mab/36892
anti-FLCN (Cell Signaling Technology, 3697) ; https://www.cellsignal.com/products/primary-antibodies/flcn-d14g9-rabbit-mab/3697
HRP-conjugated anti-rabbit IgG (Cell Signaling Technology, 7074); https://www.cellsignal.com/products/secondary-antibodies/anti-rabbit-igg-hrp-linked-antibody/7074
HRP-conjugated anti-mouse (Cell Signaling Technology, 7076) ; https://www.cellsignal.com/products/secondary-antibodies/anti-mouse-igg-hrp-linked-antibody/7076
Alexa Fluor 568-conjugated goat anti-mouse IgG (Invitrogen, A21090) ; https://www.thermofisher.com/antibody/product/Goat-anti-Human-IgG-H-L-Cross-Adsorbed-Secondary-Antibody-Polyclonal/A-21090
Alexa Fluor 488-conjugated goat anti-rabbit IgG (Invitrogen, A-11008) ; https://www.thermofisher.com/antibody/product/Goat-anti-Rabbit-IgG-H-L-Cross-Adsorbed-Secondary-Antibody-Polyclonal/A-11008
Alexa Fluor 488-conjugated goat anti-mouse IgG (Invitrogen, A-11001) ; https://www.thermofisher.com/antibody/product/Goat-anti-

Mouse-IgG-H-L-Cross-Adsorbed-Secondary-Antibody-Polyclonal/A-11001.

Eukaryotic cell lines

Policy information about cell lines and Sex and Gender in Research

Cell line source(s)	U2OS cells (ATCC, HTB-96) and HeLa cells (ATCC, CCL-2) were obtained from ATCC.
Authentication	Authentication of the cell line were performed by a Human STR Profiling Cell Authentication Service (ATCC).
Mycoplasma contamination	All cell lines are free of mycoplasma contamination.
Commonly misidentified lines (See ICLAC register)	No commonly misidentified cell lines were used

Animals and other research organisms

Policy information about studies involving animals; ARRIVE guidelines recommended for reporting animal research, and Sex and Gender in Research

Laboratory animals	4-12 months old adult zebrafish (Danio rerio) were used to obtain fertilized eggs. Assays were conducted in zebrafish embryos and larvae at 1-8 dpf.<br>Zebrafish strains: wild-type strain (TAB-5), tmem55b-KO and tmem55-KO mutants.
Wild animals	We did not use wild animals.
Reporting on sex	Assays were conducted in zebrafish embryos and larvae up to 7 dpf. At these developmental stages, sex is not yet determined
Field-collected samples	We did not use field-collected samples.
Ethics oversight	All zebrafish experiments were performed in compliance with the National Institutes of Health (NIH) guidelines for animal handling and research under NHLBI Animal Care and Use Committee (ACUC) approved protocol H-0252R5.

Note that full information on the approval of the study protocol must also be provided in the manuscript.

Flow Cytometry

Plots

Confirm that:

- [x] The axis labels state the marker and fluorochrome used (e.g. CD4-FITC).
- [x] The axis scales are clearly visible. Include numbers along axes only for bottom left plot of group (a 'group' is an analysis of identical markers).
- [x] All plots are contour plots with outliers or pseudocolor plots.
- [x] A numerical value for number of cells or percentage (with statistics) is provided.

Methodology

Sample preparation	For Annexin V apoptosis Detection, U2OS cells were washed with PBS and treated with TrypLE express enzyme (Gibco, 12605010) for less than 3 min at 37 °C. Subsequently, the cells were incubated with eBioscience Annexin V Apoptosis Detection Kit (Invitrogen, 88-8006-72) according to the manufacturer's instructions. Cells were then analyzed by flow cytometry using a BD Fortessa cytometer. For Intracellular ROS analysis, U2OS cells were washed, trypsinized and collected. Cells were diluted with H2DCFDA (20 µM; Invitrogen, C400) for 30 min and then incubated with NaAsO2 (300 µM) for 2 h, H2O2 (500 µM) for 4 h, Acrolein (200 µM) for 2 h or Spermidine (300 µM) for 4 h. After incubation, cells were washed with PBS and flow cytometry was performed by using BD LSR Fortessa.
Instrument	BD LSR Fortessa
Software	BD FACSDiva
Cell population abundance	10,000-20,000 cells were used for analysis per samples.

Gating strategy

Apoptotic/necrotic cell populations were defined first by single cell gating and followed by quadrant gating in dot plots based on Annexin V and 7-AAD staining intensity. Four populations were revealed, representing intact cells (Annexin V-negative, 7AAD-negative), early apoptotic cells (Annexin V-positive, 7AAD-negative), late apoptotic cells (Annexin V-positive and 7AAD-positive) and necrotic cells (Annexin V-negative, 7AAD-positive).

For measuring ROS production, live singlets were first gated based on their size and granularity, and then the intensity of DCFDA was displayed in histograms to represent intracellular ROS levels in cells measured.

☒ Tick this box to confirm that a figure exemplifying the gating strategy is provided in the Supplementary Information.
